# An open window: the crucial role of the gut-brain axis in neurodevelopmental outcomes post-neurocritical illness

**DOI:** 10.3389/fped.2024.1499330

**Published:** 2025-01-20

**Authors:** Victoria Ronan

**Affiliations:** Department of Pediatrics, Section of Critical Care, Children’s Wisconsin/Medical College of Wisconsin, Milwaukee, WI, United States

**Keywords:** neurocritical illness, gut-brain axis, microbiome, outcomes, pediatric neurocritical care, neurodevelopmental outcome

## Abstract

Among patients admitted to the pediatric intensive care unit, approximately 10% are discharged with a new functional morbidity. For those who were admitted with a neurocritical illness, the number can be as high as 60%. The most common diagnoses for a neurocritical illness admission include traumatic brain injury, status epilepticus, post-cardiac arrest, hypoxic ischemic encephalopathy, meningo/encephalitis, and stroke. The gut-brain axis is crucial to childhood development, particularly neurodevelopment. Alterations on either side of the bidirectional communication of the gut-brain axis have been shown to alter typical development and have been associated with autism spectrum disorder, anxiety, sleep disturbances, and learning disabilities, among others. For those patients who have experienced a direct neurologic insult, subsequent interventions may contribute to dysbiosis, which could compound injury to the brain. Increasing data suggests the existence of a critical window for both gut microbiome plasticity and neurodevelopment in which interventions could help or could harm and warrant further investigation.

## 1 Introduction

Understanding and preventing acquired morbidity at discharge after critical illness is garnering greater attention due to improved survivorship. Intensive care unit (ICU) follow-up clinics for children discharged from the pediatric intensive care unit are increasing. Further, there is a growing body of literature describing outcomes following pediatric intensive care unit (PICU) admission, particularly following admission after neurocritical illness. Alarmingly acquired functional impairment in the general PICU population is reported as high as 36% at discharge, with that number decreasing over time to approximately 26% at six months and 10%–13% at 48 months (1).

Nearly 20% of PICU admissions require neurocritical care, with the most common diagnoses being traumatic brain injury, post-cardiac arrest, status epilepticus, meningo/encephalitis, and stroke (2). Direct neurologic injury can alter neuronal communication and thus change the brain's developmental trajectory. Pediatric neuroplasticity has been established in existing literature, and gliogenesis, synaptogenesis, synaptic pruning, and myelination continue beyond 10 years of postnatal age (3, 4). However, data is limited in describing long-term neurodevelopmental outcomes following intensive care admission after direct neurologic injury. The existing studies are limited by loss to follow-up, sample size, and heterogeneity of both diagnoses and outcome measures.

There remains a lot to be understood regarding the etiology and driving factors of acquired neurodevelopmental morbidity after neurocritical illness, and particularly why some children are disproportionately affected compared to their peers. A clue may lie in the development of the gut microbiome, its role in the gut-brain axis, and its influence on neurodevelopment. The gut-brain axis is a crucial contributor to neurodevelopment, and the gut microbiome is essential to the bidirectional communication that informs neurodevelopment. Many disruptors to the gut microbiome's development and stability during critical illness and ICU admission contribute to potential dysbiosis. This interplay between gut microbiome dysbiosis and direct neurologic injury may be a factor in the differing outcomes after neurocritical illness.

## 2 Neurodevelopmental outcomes

### 2.1 Neurodevelopmental outcomes after PICU admission

All patients admitted to the PICU are at risk of acquired morbidity during critical illness. One study found that up to 10% of all patients admitted to the PICU develop a new functional disability at discharge as measured by the Pediatric Cerebral Performance Category (PCPC) and Pediatric Overall Performance Category (POPC) (5). In contrast, another study found just under 5% of general PICU patients report new morbidity. Studies exploring the development of newly acquired morbidity in general PICU admissions have identified the following risk factors: an increased baseline PCPC, longer duration of chest compressions, lower oxygen saturations, the requirement of vasoactive medications, longer duration of invasive mechanical ventilation and longer stays in the PICU (6–8).

Patients admitted to the PICU with neurocritical illness are at an even higher risk of acquired morbidity compared to those with non-neurocritical illness. As many as 61% of children admitted to the PICU with neurocritical illness have a PCPC of 4–6 at discharge, consistent with severe disability, persistent vegetative state, or death (9, 10).

Outcomes are reported as worse in children admitted with stroke, cardiac arrest, and status epilepticus compared to traumatic brain injury (TBI) (9–11). TBI has a consensus protocol for management based on the severity of injury tier, while no such consensus exists in the management of other domains of neurocritical care. It is widely accepted that a protocolized approach to treating TBI that guideline adherence is associated with improved outcomes and thus may be confounded here. Vavilala et al. demonstrated for every 1% increase in adherence to guidelines in patients with TBI, there was a 1% decrease in unfavorable outcomes at discharge (12). Williams et al., looking at outcomes of children aged greater than or equal to 3 years admitted to the PICU with TBI, stroke, an infectious or inflammatory disorder, or hypoxic-ischemic injury, 56% of children with acquired brain injury reported sleep-wake disturbance and noted as severe in 46%. In this study, older median age and the presence of preadmission chronic medical conditions were significantly associated with sleep-wake disturbance at discharge from the PICU (13).

### 2.2 Neurodevelopmental outcomes after TBI

Children with severe traumatic brain injury suffer worse outcomes compared to children who suffer mild to moderate TBI. Children with mild to moderate TBI may continue to follow a developmental trajectory that, although within the normal range, is lower than their peers. Children with severe TBI, however, do not follow a typical developmental trajectory, with disparities becoming more evident over time (14). A meta-analysis examining the effects of injury severity and time since injury demonstrated children with moderate TBI performed more poorly than those with mild TBI but considerably better than those with severe TBI. Children in the moderate TBI group had a modest recovery of intellectual function and attention; however, two years post-injury had not caught up developmentally to demographically matched peers. Children with severe TBI were left with significant and persistent impairments with little recovery of function and did not make appropriate developmental gains. This disparity in recovery left them far behind their peers. Notably, younger age at injury was also associated with larger divergences from peers (15).

A Melbourne study of 48 children with TBI noted that increased severity of TBI was linked with a more significant impact on global functioning with significant differences in IQ across the groups compared to both each other and the control group. Preinjury adaptive function was noted to be the strongest predictor of post-injury performance. Additionally, while educational outcomes were variable, this may have been impacted by socioeconomic factors in conjunction with injury (16). Some functional domains may be more vulnerable than others. Across all severity groups and remaining post-injury, visual perceptual functioning may be the neurocognitive domain least affected. Fluency and problem-solving appear most vulnerable to disruption across all severity groups (15).

Children with abusive head trauma may have worse outcomes in gross motor, personal/social, and problem-solving aspects of development (14). A systematic review found that preinjury familial environment and styles are linked to TBI outcomes. The pre- and post-injury family environment predicted chronic changes, suggesting that the disruption of illness, injury, and other adverse events would either be buffered or exacerbated by the familial or home environment (17).

Depressive symptoms are more common post-TBI compared to noninjured or orthopedic injury populations. Risk factors for the development of depression and/or depressive symptoms may be injury-related (such as brain lesions or pain) and noninjury-related (such as older age at injury, lower SES) (18). The prevalence of depressive symptoms in children injured at age 12 years or older was reported as high as sixfold that of children who sustained an injury at age less than 9 years (19). The presence of self-reported depressive symptoms is a predictor of poor sleep quality in adolescents with mild, moderate, and severe TBI, in addition to poorer school functioning at 12- and 24 months post-injury (20).

In children aged 3–18 years, just over two-thirds experienced clinically significant sleep-wake disturbance associated with executive function outcomes after discharge (21). A meta-analysis found sleep disturbance to be highly prevalent in the acute phase of recovery in children, within the first 1 week to 1 month, and improved as time since injury increased. Approximately 20% of children reported sleep disturbance at three months post-TBI. Older age at the time of injury was related to worse sleep outcomes (22).

### 2.3 Neurodevelopmental outcomes after stroke

Pediatric stroke can be divided into perinatal stroke (less than or equal to 28 days old) and childhood stroke (occurring between 28 days and 18 years). Perinatal stroke is reported in 1 in 2,300 live births, whereas childhood stroke is reported in 1–13 per 1,000 (23–25). This categorization is important as there are differences between the two age groups in etiology, risk factors, presentation, and outcomes.

Data on outcomes after pediatric stroke show wide variance with no clear consensus in literature, likely due to variances in location of insult, etiology, among other factors. The percentage of children who are left with moderate to severe disability following stroke is reported as anywhere between 30% and 60%. Studies in Europe report a moderate to severe disability (as defined by modified Rankin score > 2) in up to 1/3 of patients following pediatric stroke (26, 27). A Canadian registry, which included neonatal and childhood stroke, reported moderate to severe deficits in 1/3 of cases (28). A single-center study in London reported poorer outcomes interfering with daily life in up to 60% of patients (29). However, a study from the Netherlands reported no severe disability in a population of 27 patients (30).

Outcomes following pediatric stroke most consistently are reported to be associated with age at stroke, location of stroke, etiology (hemorrhagic vs. ischemic), and the development of epilepsy following injury. Age at injury has conflicting associations across published studies. While some reports note a younger age at the time of stroke to be associated with a worse outcome, some studies do not report a poorer outcome with younger age, irrespective of lesion size or location in domains of cognitive flexibility, processing speed, and verbal learning (29, 31–35). Early childhood stroke had significantly worse cognitive outcomes compared to neonatal or late childhood stroke (36).

A re-analysis of the vascular effects of infection in pediatric stroke (VIPS study) found an association between larger infarct volume and younger age at stroke with poorer outcomes. However, the authors did note the strength of those associations was limited (37). The location of the infarct is also tied to the outcome. Infarcts that interrupted key networks had a disproportionate impact on outcomes following acute ischemic stroke. Infarcts involving uncinate fasciculus, angular gyrus, insular cortex, or that extended from cortex to subcortical nuclei were significantly associated with worse outcomes in the VIPS study (37). Compared to survivors of anterior circulation acute ischemic stroke, children with posterior circulation strokes had more favorable outcomes but were also more likely to receive anti-thrombotic agents (38). A single institution study in Colorado noted acute cerebral arteriopathy and elevated d dimer levels were identified as prognostic factors for poor outcomes (39).

Neuropsychological problems, learning difficulties, and mental health issues are among the commonly reported long-term issues following pediatric stroke. Learning disabilities and lower IQ compared to siblings or healthy counterparts have also been reported (29, 32). A pediatric stroke registry in Switzerland found processing speed and auditory short-term memory were particularly affected in approximately 75% of survivors (31). One study following outcomes ten years after childhood stroke reported that 80% of patients had a complete recovery or mild deficit on a modified ranking score, but over 1/4 of them also self-reported mental health issues compared to 5% of healthy counterparts aged three to 17 years old who report anxiety and depression at the time of this study. It is important to note that in the general population, self-reporting of mental health issues such as depression and anxiety have trended up significantly following the COVID-19 pandemic, and while this is not the scope of this review, it does highlight that studies of mental health issues following neurocritical illness, should be taken within the context of public health data collected concurrently (40, 41).

Measuring outcomes following pediatric stroke presents many challenges. The complex interaction between normal childhood development of the brain and recovery mechanisms following a stroke leads to an evolving neurologic exam and functional ability assessment throughout childhood. This represents a unique challenge to studying stroke outcomes in children compared to their adult counterparts (42–44). Another limitation to measuring outcomes in pediatric stroke is using the pediatric stroke outcome measure, a quantitative validated measure of outcome in pediatric stroke based on five domains of neurologic function. It may overestimate the consequences of pediatric stroke compared to the modified Rankin Scale as the latter focuses on function rather than neurologic impairment. The heterogeneity of study populations and the lack of consensus in outcome measurement make data difficult to compare between studies.

### 2.4 Neurodevelopmental outcomes after meningo/encephalitis

Inflammatory changes due to central nervous system infections cause brain injury and lead to varying outcomes. Mortality following meningoencephalitis has been reported anywhere between 5% and 30%. Of those who survive, up to 50% have neurologic sequelae, and approximately 40% report some degree of developmental delay (45, 46). Earlier age at diagnosis and age under 12 months have been linked to poorer neurologic outcomes. Additional risk factors include thrombocytopenia, need for mechanical ventilation, delayed presentation, anemia, underweight, or seizures at presentation (47–49).

The most common acquired morbidities reported are hearing loss, developmental delay, behavioral problems, difficulties with sleep, and a decline in school performance (50–53). One study of Dutch children reported that children with bacterial meningitis were twice as likely to repeat a school year as healthy peers or siblings (54). School problems, difficulty with educational achievements, and difficulty concentrating, in addition to lower rates of economic self-sufficiency later in life, are reported among survivors of central nervous system infection (54, 55). A study of survivors of childhood bacterial meningitis reported that 41% had some developmental delay, with almost one-third reporting psychomotor delay. Residual cognitive dysfunction has been reported in 5% of children at three-year follow-up in a study of children with enterovirus encephalitis (46, 56).

The etiology of encephalitis and geographical location are also important predictors of outcome (53, 57). While the etiology of encephalitis is an important predictor of outcome, the epidemiology is geographically dependent. A 2021 study comparing outcomes among children with bacterial meningitis in Finland, Angola, and Latin America found significant geographic variability in outcomes, likely due to a combination of public health and individual factors (49). The study highlighted those children with anemia who were underweight, presented late, or had seizures as part of their presentation had worse outcomes. Outcomes with less reported morbidity observed in countries like Finland may be attributed to multifactorial influences, including public health infrastructure and individual health characteristics.

### 2.5 Neurodevelopmental outcomes after status epilepticus

Convulsive status epilepticus has its highest incidence during the first three years of life. This period represents a critical window of neurological development, thus increasing susceptibility to early neurologic insults (58–61). Following at least one episode of convulsive status epilepticus, whether a prolonged febrile seizure or non-febrile convulsive status, developmental impairments evident at six weeks post-acute event remain significant at 12 months (62). Within the first year after a prolonged febrile seizure, affected children often present with recognition memory impairments (63).

Despite scoring within clinically normal ranges, children who had experienced at least one episode of prolonged febrile seizure within the first three years of life underperformed on developmental assessments compared to controls (63). Mortality rates for convulsive status epilepticus can be as high as 20% (58, 64–67). For survivors, cognitive and motor disabilities are reported in up to 56% of cases (68–71). Factors associated with worse outcomes following convulsive status epilepticus include a higher Pediatric Cerebral Performance Category (PCPC) at baseline, diazepam-resistant convulsive status epilepticus, conversion to refractory or super refractory status epilepticus, the need for invasive mechanical ventilation, abnormal electroencephalogram (EEG) background, and non-convulsive status epilepticus (72).

While electrographic status epilepticus (ESE) and status epilepticus (SE) may serve as clinical indicators of brain dysfunction, they also contribute to secondary injury. This is associated with worse adaptive behavioral global composite scores, poorer long-term outcomes, lower quality of life, and an increased risk of epilepsy (73). One study involving 200 children undergoing continuous EEG monitoring in the PICU found that ESE was linked to an increased risk of mortality and a worsening of the PCPC at discharge, in contrast to electrographic seizures (ES), which showed no increased risk of mortality or PCPC worsening (74).

### 2.6 Neurodevelopmental outcomes after cardiac arrest

Within the last 20 years, there has been a shift from thinking of pediatric cardiac arrest as a futile medical condition to one that has been the focus of many studies intending to improve mortality. Recent literature shows that survival to hospital discharge rates and pediatric out-of-hospital cardiac arrest (8.2%–8.6%) approaches that of adults (8.8%) (75, 76). Despite advances in resuscitation techniques and post-resuscitation care leading to higher survival rates, survivors of pediatric cardiac arrest can be left with significant neurological sequelae that are not well elucidated in current literature.

Approximately 20,000 children sustain cardiac arrest annually in the United States. Of these, 15,000 children are resuscitated for in-hospital cardiac arrest (IHCA). When compared to out-of-hospital cardiac arrest (OHCA), pediatric IHCA has higher rates of survival (77–80). Among children who survive pediatric cardiac arrest, neurologic injury is the leading cause of morbidity and mortality. Of patients who survive IHCA, as few as 22% have been reported to have good neurologic outcomes (81). Patients who survive cardiac arrest but are discharged with poor neurologic status carry a significant risk of death, as high as 54% (82).

Outcomes following cardiac arrest are largely dependent on the location of cardiac arrest. Children who have an IHCA tend to receive more rapid resuscitation and, thus, less injury to the brain. Many studies have explored long-term neurologic outcomes in children who have survived hospital cardiac arrest. Still, due to the nature of pediatric OHCA, these studies have been limited by small sample size, single-site studies, narrow age range, or limited number of etiologies (83–89).

In studies examining long-term outcomes following OHCA by age group, there have been inconsistent findings. Some studies suggest that older age at the time of cardiac arrest and follow-up is associated with worse outcomes in neuropsychological and neurobehavioral testing (86). One such study examining neurobehavioral outcomes at 12 months in children who survived OHCA found that older children tended to have significant morbidity in all functional domains and sub-domains, with motor and daily living skills most affected (90). Other studies found that while overall favorable neurologic outcomes were reported to be over a wide range (10%–71%) more adolescents tended to have a favorable neurologic outcome at follow-up compared to infants (91, 92). At 24 months, OHCA survivors have worse intellectual functioning compared with normal data across various neuropsychological tests, including total IQ, verbal IQ, selective attention, sustained attention, processing speed, verbal memory, and cognitive flexibility (92, 93).

Prognostication for post-cardiac arrest neurologic outcome presents many challenges. Certain risk factors, such as the use of vasoactive-inotropic drugs before cardiac arrest, a previous PCPC scale score greater than 2, an underlying hemato-oncologic disease, and the total duration of CPR, are associated with poorer outcomes (81). Trauma or neurologic illness as etiology for IHCA and lactic acidemia more than 24 h following ROSC were found to be associated with a worse long-term neurological outcome (94). Blood-based brain injury biomarkers may be associated with outcomes following pediatric cardiac arrest as reported in several small observational studies and one large prospective multi-center cohort study The identified biomarkers of brain injury include glial fibrillary acidic protein (GFP), ubiquitin carboxyl-terminal esterase L1 (U CH-L1), Tau protein, and especially neurofilament light (NfL-1), are associated with death and or a more unfavorable outcome at 12 months post cardiac arrest. While concentrations of all biomarkers were higher in children with an unfavorable outcome compared to those with a favorable outcome at 1 year, both NfL1 and UCH-L1 had higher overall accuracy and reliability for 1 year post arrest outcomes (95–97).

Like other studies of long-term outcomes following pediatric neurocritical illness, studies describing outcomes after pediatric cardiac arrest are limited by the heterogeneity of the study population, loss to follow-up, and lack of consensus on outcome measures often leading to conflicting data. It is an interesting finding within this cohort that older children tend to have worse outcomes following cardiac arrest compared to younger counterparts which suggests there may be either some previously unidentified vulnerability and or a need for more sensitive testing for our younger populations that has not previously been explored that differentiates the neuro trajectory for older children Table 1.

**Table 1:** This table summarizes data presented on incidence, risk factors and notable outcomes in pediatric neurocritical illness. .

	Incidence	Risk factors	Notable outcomes
Post-PICU admission	Up to 10% of all PICU admissions have acquired neurologic morbidity (5–8) Up to 61% children with any neurocritical illness have a new neurologic disability (9, 10)	• Higher baseline PCPC (5–8) • Longer duration of chest compressions (5–8) • Lower oxygen saturations (5–8) • Use of vasoactive medications (5–8) • Longer duration of invasive mechanical ventilation (5–8) • Longer length of PICU stay (5–8)	New functional disability as measured by PCPC and/or POPC (5, 9, 10) Disturbance in sleep wake cycle (13)
Post TBI	Children with severe TBI have worse outcomes compared to those with mild-moderate TBI	• Conflicting evidence re: age at injury • Abusive head trauma (14) • Severe TBI (15) • Lower pre injury adaptive function (16) • Socioeconomic factors play some role (16)	Worse outcomes in gross motor, personal/social, and problem-solving aspects of development. (14)
Post Stroke	30–60% (26–30)	• Conflicting evidence re: age at injury • Location of stroke (37) • Etiology (hemorrhagic versus ischemic) (26, 27, 29, 31, 35, 37) • Development of epilepsy post stroke (29, 31, 35, 37)	Neuropsychological problems, learning difficulties, and mental health issues (29, 31, 32, 40, 41) Depressive symptoms (18) Disruption of sleep wake cycles (21, 22)
Post Meningo-/Encephalitis	Among survivors up to 50% have neurologic sequelae and 40% have developmental delay (45, 46)	• Younger age at diagnosis (47–49) • Thrombocytopenia and/or anemia (47–49) • Need for mechanical ventilation (47–49) • Delayed presentation (49) • Depressed level of consciousness (51) • Seizures at presentation (47–49) • Underweight for age (49) • Etiology and geographic location (53, 57)	Hearing loss (50) Developmental delay (51–54) Behavioral problems (51–54) Difficulties with sleep (51–54) Decline in school performance (51–54)
Post Status Epilepticus	Up to 56% acquired neurologic morbidity among survivors (68–71)	• Prolonged febrile seizure before age 3 years (72) • Higher PCPC at baseline (72) • Diazepam-resistant convulsive SE (72) • Conversion to refractory or super refractory SE (72) • Need for invasive mechanical ventilation (72) • Abnormal EEG background (72) • Non-convulsive status epilepticus (72)	Recognition and memory impairments (63) Increased risk of epilepsy (73) Worse performance on adaptive behavioral global composite scores (73) Lower quality of life (73)
Post Cardiac Arrest	Up to 54% of survivors discharged with poor neurologic status (82)	• Out of hospital cardiac arrest (83–89) • Use of vasoactive drugs prior to arrest (81) • Premorbid PCPC > 2 (81) • Duration of CPR (81) • Trauma or neurologic illness as etiology of arrest (94) • Hemato-oncologic disease (94) • Elevated biomarkers: (94–97) − lactic acidemia pre arrest − elevated NfL-1 and UCH-L1 post arrest	All functional domains with motor domains more affected (90) Worse intellectual functioning (92, 93)

It is important to note that the current literature is limited by heterogeneity of study populations, lack of consensus in measures of outcome data, and loss to follow-up. CPR, cardiopulmonary resuscitation; EEG, electroencephalogram; NfL-1, neurofilament light; PCPC, Pediatric Cerebral Performance Category; PICU, pediatric intensive care unit; POPC, Pediatric Overall Performance Category; SE, status epilepticus; TBI, traumatic brain injury; UCH-L1, ubiquitin carboxyl-terminal esterase L1.

## 3 The development of the gut microbiome and potential factors in critical illness contributing to dysbiosis

From infancy through adolescence, physiologic milestones and environmental changes contribute to the development of the gut microbiome from that of a child to that of an adult. Throughout childhood and adolescence, the gut microbiome increases in both diversity and stability. At birth the maternal microbiome seeds the infant gut microbiome, with approximately half of strains of species shared between mother and infant (98). After birth, the gut microbiome is heavily influenced by the mode of delivery, environmental factors at birth and postnatally, type of milk consumption, and exposure to medications. With the significant change in diet accompanying weaning, the proteobacteria in the gut microbiome become much less abundant. Over time shared strains between mother and offspring decrease from 50% at birth to rates seen among household members by age 3 years (98–102).

Further shifts in the microbiota occur in children with the expansion of the social environment during school attendance, exposure to pets, and dietary changes (101, 102). Although the developing adolescent gut microbiome has not yet reached that of an adult, there is an overall shift toward a decreased abundance of aerobes and facultative anaerobes with a concomitant increase in anaerobic species, which is characteristic of an adult microbiome (103). While specific microbiotal populations and the exact timing of shifts are not yet known, it is clear there are environmental and physiologic triggers that incite change.

Dysbiosis is a disruption leading to an alteration in the microbiota, thereby changing the composition and metabolic profile of the microbiome. Either because of presenting illness or exposure to medications and the environment within the ICU, critically ill patients experience significant alterations to their microbiome. Common medications during ICU admission, such as vasopressors, antibiotics, and gastric acid suppressors, change the gut microbiome. Within the ICU, exposure to surgical procedures, changes to mode or type of feed, invasive mechanical ventilation, and the presence of nosocomial pathogens also contribute to a shift within the microbiota (104–106). Most data on ICU-related dysbiosis are based on studies of adult patients; few studies examine this phenomenon in children. In critically ill children and adults, the limited existing data notes a loss of diversity, a loss of body site specificity, and a higher abundance of pathogens within the gut microbiome during ICU admission (107, 108).

Studies attempting to characterize microbiome changes in critical illness have been limited by the heterogeneity of their patient populations and single-time point/single-site sampling. Once ICU dysbiosis occurs, it may persist as a maladaptive state with important ramifications for host health, such as chronic inflammation, susceptibility to secondary infections, and alteration of development (109–112).

Recent studies evaluating the microbiome in traumatic brain injury have reported important findings. Changes in taxonomic diversity occur as early as 24 h after injury and persist at 28 days in mouse models (104). In adults, gut microbiome disruption may persist for years after acquiring a moderate or severe TBI. The relevance of changes in gut microbiota to outcomes after neurologic injury is not clear yet in clinical practice. In a murine model, the depletion of intestinal microbiota was consistent with a neuroprotective effect following TBI (113). Among human and murine studies, decreases in richness and commensal diversity lead to decreased bacteria that produce short-chain fatty acids (SCFA) within the gut (105, 114–116). SCFAs are critical to blood-brain barrier permeability, microglial polarization and function, and neurogenesis (117). Additionally, SCFAs are crucial for the normal development of the hypothalamus-pituitar*y* axis (118, 119). When considering the risk factors for worse neurologic outcomes across multiple etiologies of neurocritical illness, many, if not all, of these can contribute to dysbiosis.

## 4 The importance of the gut-brain axis on neurodevelopment

The bi-directional communication of the gut-brain axis is crucial for neurodevelopment. Signaling from the brain to the gut via the afferent fibers of the vagus nerve influences motor, sensory, and secretory functions of the gastrointestinal tract. The efferent vagal nerve fibers communicate from the gut to the brain. Interleukin signaling molecules and bioactive metabolites (such as serotonin, dopamine, noradrenaline, acetylcholine, and GABA) are produced by the gut microbiota and can function as neurotransmitters (120–123). Alteration of the metabolic profile of the gut microbiome and resulting dysbiosis can influence the communication between the central nervous system (CNS) and enteric nerve, the immune function of the brain, the inflammation of the CNS, and the function and integrity of the blood-brain barrier.

**Figure 1:**
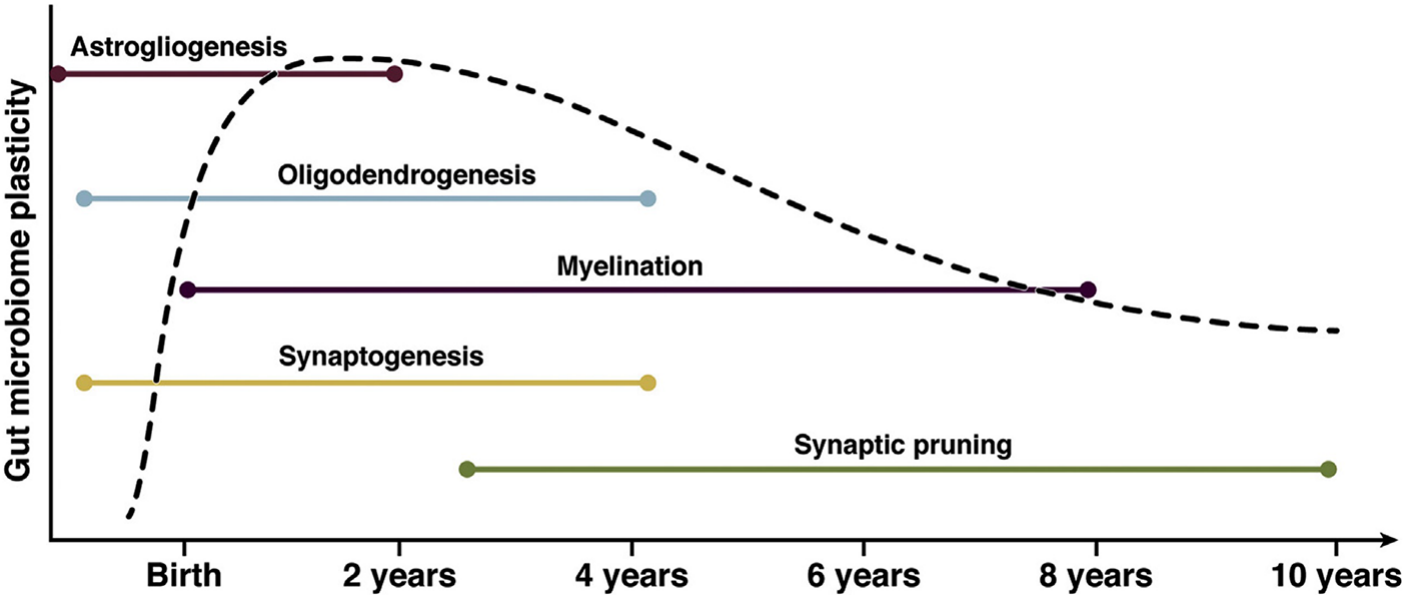
The parallel neurodevelopment and microbiome development during childhood. This graph demonstrates periods of highest microbiotal plasticity and growth occurring concurrently with neurodevelopment. The periods of highest neuronal growth and gut microbiome plasticity reflect potentially vulnerable windows for modulation of the gut-brain axis. (112) Ronan, 2021, Gastroenterology, permission for use granted by Elsevier. Copyright Elsevier, 2021.

The process of neurodevelopment occurs in parallel with the development of the gut microbiome. (Figure 1) While neuronal migration and neurogenesis are typically fetal processes, gliogenesis, synaptogenesis, myelination, and synaptic pruning continue through adolescence. Astrogliogenesis begins prenatally and continues to 2 years postnatal age. Astrocytes shape neuronal circuits in the developing brain by coordinating the formation and function of synapses, the survival of neurons, and the guidance of axons. Synaptogenesis begins prenatally and continues through age 4 years. The process of synaptic pruning occurs from ages 3 to 10 years. Oligodendrocytes are responsible for the generation and maintenance of myelination. The process of myelination continues from birth to 8 years, with approximately 80% of myelination occurring between 2 and 3 years of age. Microglia are an integral neurodevelopmental factor crucial in developing innate immunity, neuroprotection, and synaptic pruning continuing into adolescence (4, 120, 122).

Gut dysbiosis is associated with disordered neurodevelopment. In human studies, dysbiosis has been associated with disorders of expressive and receptive language, attention deficit hyperactivity disorder (ADHD), and increases in fear reactivity and oppositional defiant behavior (124). Higher alpha diversity in the first year of life is linked to poorer results on early learning scores, visual reception scales, and expressive language scales (125). A study of the gut microbiota in Serbian children found those with neurodevelopmental disorders tended to have fewer SCFA butyrate-producing taxa and greater *Clostridium* spp. Studies have also shown dysregulation mechanisms of dopamine, serotonin, and norepinephrine as a result of dysbiosis in children with neurodevelopmental disorders (126–128). Children with autism spectrum disorder (ASD) have a higher abundance of *Bacteroides* and a lower abundance of Firmicutes. Additionally, late-onset ASD has been associated with increased levels of *clostridium* spp within the gut microbiome (129). Mice who had fecal transplants from human donors with ASD displayed ASD-like behaviors and were later found to have ASD-relevant genes. These findings promote the theory that the gut microbiota can regulate behavior through neuroactive metabolites via the gut-brain axis (130). This theory was further supported in studies where children with autism received fecal matter transplants from typically developing peers and subsequently showed improved social skills and adaptive behaviors (131–133).

The gut microbiota has also been implicated in the development of neuropsychiatric disease. Long-term treatment with the probiotic *L. rhamnosus* led to decreased levels of stress-induced corticosteroids, depressive symptoms, and anxiety in a mouse model (134). A germ-free murine model displayed increased motor activity and a lack of appropriate anxiety-like behavior in tests compared to specific pathogen-free counterparts. This behavioral finding correlated with an increase in neurotransmitters and dopamine receptors (135). Further, the Flemish Gut Flora Project found that features of the microbiome correlate with host quality of life and depression. Butyrate producing bacteria, such as *Faecalibacterium* and *Coprococcus* were associated with higher quality of life indicators. *Coprococcus* spp. were noted to be depleted in depression, even accounting for confounders such as antidepressants. Additionally, they commented on the potential for microbial synthesis of dopamine metabolites to contribute to higher quality of life and a role for microbial *γ*-aminobutyric acid production in depression. This study importantly highlights how neurally active metabolites synthesized by the gut flora act as messengers of gut brain axis in the development of health and disease (136).

## 5 A critical window to help or hurt

Throughout childhood, the developing brain undergoes a series of changes to cellular composition, neuronal circuitry, and blood flow that represent a dynamic environment and a vulnerability to injury and recovery mechanisms (137). The Kennard principle proposed, in the 1930s, after studies investigating the age of motor impairment after brain injury and monkeys that the earlier a brain injury occurs, the more likely there are compensatory mechanisms that would reduce negative effects and lead to improved outcomes compared to older populations (138). However, in the 1950s, Hebb put forth a competing hypothesis suggesting a selective vulnerability for some patients who had brain injury earlier in life compared to adulthood (139). Since these early hypotheses, numerous studies have added to a far more nuanced picture of how age at neurologic injury, the size of the insult, the type of insult, the location of the insult, and baseline neurologic function may influence outcomes (140–143).

Current evidence suggests children who sustain diffuse brain injuries early in life are more vulnerable to long-lasting impairment compared to those injured later in life; however, there may be more than one critical developmental period that's associated with a heightened risk for worse outcomes. This may be the reason for an increased risk to older populations past school age, not just neonates and infants (144–147).

Evidence exists for a critical window for microbiotal modulation, which, if missed, reduces the impact of interventions. Cowan et al. first proposed the existence of a critical window for gut microbiota and development during which expected and/or unexpected environmental exposures may have varying effects (148). In a germ-free mouse model recolonized with “normal” gut flora at varying ages, found the window most sensitive to recolonization was before and at weaning, but not after. This was further demonstrated by Lynch et al. who demonstrated *in vivo* that microbiota depletion early in life had long-standing effects (149). In this study, the disruption of gut microbiota, especially during weaning, affected circulating immune cells, microglial function and morphology, and myelin gene expression. These changes to neurophysiology and development persisted into adolescence and adulthood in the form of anxiety-related behaviors. Callaghan, in response to the critical window proposal by Cowan et al, noted importantly, though unlikely the most important factor, the gut microbiome may play a significant role in the development of health and disease (150). This critical window hypothesis is supported in humans by Slykerman et al. whose work demonstrated that exposure to antibiotics within the first year of life, but not after, negatively impacted cognitive development (151).

**Figure 2:**
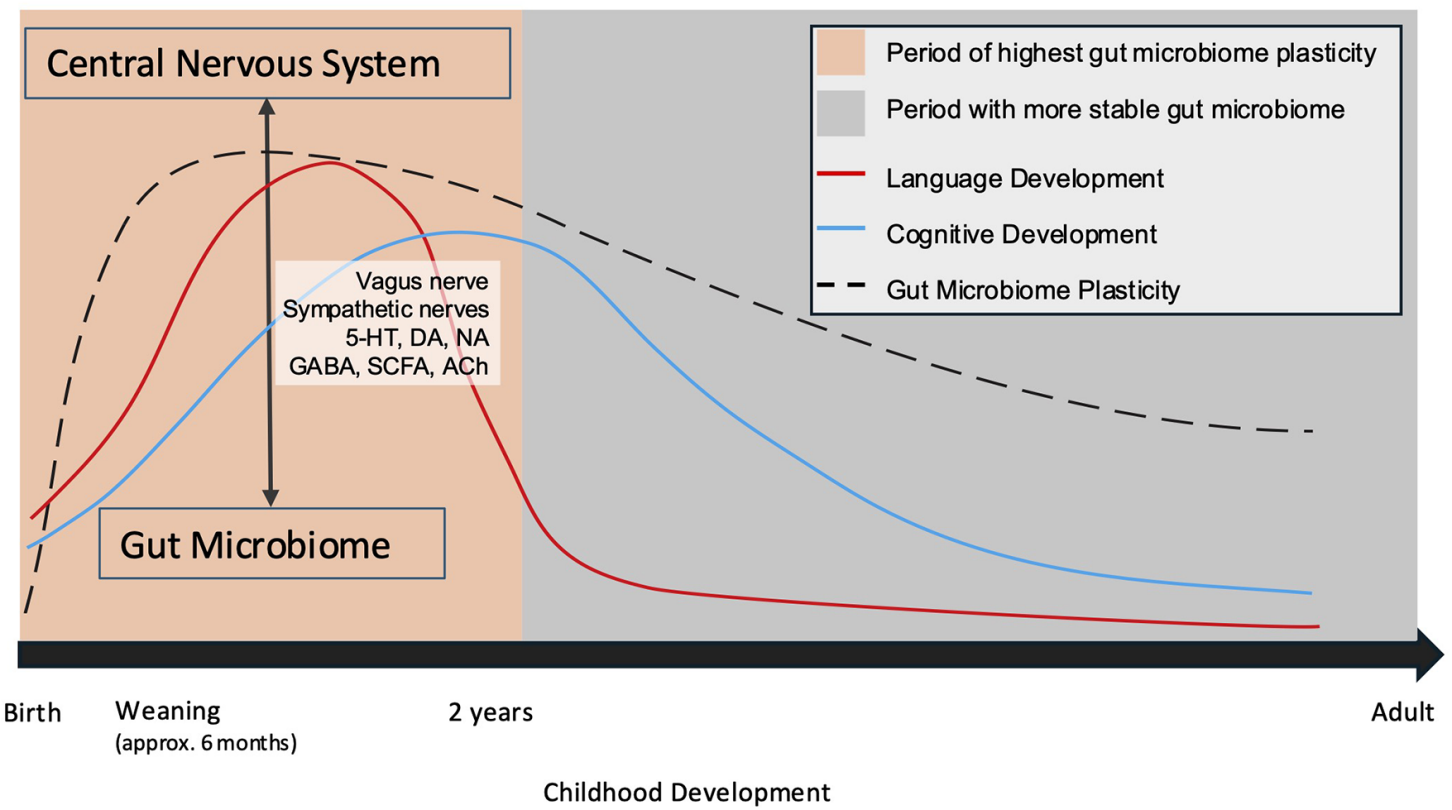
Neurodevelopment occurs chronologically and in parallel with the development of the gut microbiome. (109, 145) Periods during which the gut microbiota undergo growth and experience plasticity are critical windows during which the gut-brain axis may be crucially altered. 5-HT, serotonin; DA, dopamine; NA, noradrenaline; GABA, Gamma-aminobutyric acid; SCFA, short chain fatty acids; Ach, acetylcholine.

A window of opportunity for gut microbiome modulation exists within the first two years of life, mirroring the time during which crucial milestones of childhood development are occurring (152). (Figure 2) During this period, the gut microbiota reflects the previously experienced exposome. As such, disruption to the bi-directional communication of the gut-brain axis could have long-standing effects, which have not yet been characterized. The worse outcomes that tend to be described in younger patients may reflect primary insult and secondary injury leading to gut dysbiosis and altering the trajectory of development in such a manner as to compound injury. Similarly, there may be taxa within the gut that could afford a protective effect.

Studies characterizing gut flora changes with injury, the duration of changes, and children's subsequent developmental outcomes after neurocritical illness are warranted. As a field, we should learn from previous studies and move toward collaboration to grow our knowledge base. A more unified approach to assessing neurodevelopmental outcomes and tracking acquired functional morbidities is called for. Collaboration across multiple sites is crucial to gain the statistical power necessary to draw widely applicable conclusions from gut microbiome data. Hurdles that lie ahead include a variance in follow-up rates across different centers, the availability of follow-up care, and the cost associated with microbiome research.

## Data Availability

The original contributions presented in the study are included in the article/Supplementary Material, further inquiries can be directed to the corresponding author.

## References

[1] Ong C Lee JH Leow MK Puthucheary ZA. Functional outcomes and physical impairments in pediatric critical care survivors: a scoping review. Pediatr Crit Care Med. (2016) 17(5):e247–59. 10.1097/PCC.0000000000000706 27030932

[2] Wainwright MS Hansen G Piantino J. Pediatric neurocritical care in the 21st century: from empiricism to evidence. Curr Opin Crit Care. (2016) 22(2):106–12. 10.1097/MCC.0000000000000288 26863153

[3] Hoffmann A Ziller M Spengler D. Childhood-onset schizophrenia: insights from induced pluripotent stem cells. Int J Mol Sci. (2018) 19(12):3829. 10.3390/ijms19123829 30513688 PMC6321410

[4] Menassa DA Gomez-Nicola D. Microglial dynamics during human brain development. Front Immunol. (2018) 9:1014. 10.3389/fimmu.2018.01014 29881376 PMC5976733

[5] Bone MF Feinglass JM Goodman DM. Risk factors for acquiring functional and cognitive disabilities during admission to a PICU. Pediatr Crit Care Med. (2014) 15(7):640–8. 10.1097/PCC.0000000000000199 25072478

[6] Ebrahim S Singh S Hutchison JS Kulkarni AV Sananes R Bowman KW Adaptive behavior, functional outcomes, and quality of life outcomes of children requiring urgent ICU admission. Pediatr Crit Care Med. (2013) 14(1):10–8. 10.1097/PCC.0b013e31825b64b3 23132399

[7] Pinto NP Rhinesmith EW Kim TY Ladner PH Pollack MM. Long-term function after pediatric critical illness: results from the survivor outcomes study∗. Pediatr Crit Care Med. (2017) 18(3):e122–e30. 10.1097/PCC.0000000000001070 28107265

[8] Namachivayam P Taylor A Montague T Moran K Barrie J Delzoppo C Long-stay children in intensive care: long-term functional outcome and quality of life from a 20-yr institutional study. Pediatr Crit Care Med. (2012) 13(5):520–8. 10.1097/PCC.0b013e31824fb989 22805156

[9] Nenadovic V Stokic M Vukovic M Dokovic S Subotic M. Cognitive and electrophysiological characteristics of children with specific language impairment and subclinical epileptiform electroencephalogram. J Clin Exp Neuropsychol. (2014) 36(9):981–91. 10.1080/13803395.2014.958438 25264145

[10] Nishiyama M Nagase H Tanaka T Fujita K Kusumoto M Kajihara S Short and long-term outcomes in children with suspected acute encephalopathy. Brain Dev. (2016) 38(8):731–7. 10.1016/j.braindev.2016.02.011 26952815

[11] Nishiyama M Nagase H Tanaka T Fujita K Maruyama A Toyoshima D Demographics and outcomes of patients with pediatric febrile convulsive status epilepticus. Pediatr Neurol. (2015) 52(5):499–503. 10.1016/j.pediatrneurol.2015.02.001 25769239

[12] Vavilala MS King MA Yang JT Erickson SL Mills B Grant RM The pediatric guideline adherence and outcomes (PEGASUS) programme in severe traumatic brain injury: a single-centre hybrid implementation and effectiveness study. Lancet Child Adolesc Health. (2019) 3(1):23–34. 10.1016/S2352-4642(18)30341-9 30473440 PMC6301024

[13] Williams CN Hartman ME McEvoy CT Hall TA Lim MM Shea SA Sleep-wake disturbances after acquired brain injury in children surviving critical care. Pediatr Neurol. (2020) 103:43–51. 10.1016/j.pediatrneurol.2019.08.010 31735567 PMC7042044

[14] Keenan HT Clark A Holubkov R Ewing-Cobbs L. Longitudinal developmental outcomes of infants and toddlers with traumatic brain injury. JAMA Network Open. (2023) 6(1):e2251195. -e. 10.1001/jamanetworkopen.2022.51195 36648943 PMC9856699

[15] Babikian T Asarnow R. Neurocognitive outcomes and recovery after pediatric TBI: meta-analytic review of the literature. Neuropsychology. (2009) 23(3):283–96. 10.1037/a0015268 19413443 PMC4064005

[16] Catroppa C Anderson VA Morse SA Haritou F Rosenfeld JV. Outcome and predictors of functional recovery 5 years following pediatric traumatic brain injury (TBI). J Pediatr Psychol. (2008) 33(7):707–18. 10.1093/jpepsy/jsn006 18296728

[17] Fischer JT Bickart KC Giza C Babikian T. A review of family environment and neurobehavioral outcomes following pediatric traumatic brain injury: implications of early adverse experiences, family stress, and limbic development. Biol Psychiatry. (2022) 91(5):488–97. 10.1016/j.biopsych.2021.08.012 34772505

[18] Peterson RL Connery AK Baker DA Kirkwood MW. Preinjury emotional-behavioral functioning of children with lingering problems after mild traumatic brain injury. J Neuropsychiatry Clin Neurosci. (2015) 27(4):280–6. 10.1176/appi.neuropsych.14120373 26258490

[19] Max JE Keatley E Wilde EA Bigler ED Schachar RJ Saunders AE Depression in children and adolescents in the first 6 months after traumatic brain injury. Int J Dev Neurosci. (2012) 30(3):239–45. 10.1016/j.ijdevneu.2011.12.005 22197971 PMC3322312

[20] Tham SW Fales J Palermo TM. Subjective and objective assessment of sleep in adolescents with mild traumatic brain injury. J Neurotrauma. (2015) 32(11):847–52. 10.1089/neu.2014.3559 25707446 PMC4449620

[21] Williams CN McEvoy CT Lim MM Shea SA Kumar V Nagarajan D Sleep and executive functioning in pediatric traumatic brain injury survivors after critical care. Children (Basel). (2022) 9(5):748. 10.3390/children9050748 35626925 PMC9139390

[22] Djukic S Phillips NL Lah S. Sleep outcomes in pediatric mild traumatic brain injury: a systematic review and meta-analysis of prevalence and contributing factors. Brain Inj. (2022) 36(12-14):1289–322. 10.1080/02699052.2022.2140198 36413091

[23] Cooper AN Anderson V Hearps S Greenham M Ditchfield M Coleman L Trajectories of motor recovery in the first year after pediatric arterial ischemic stroke. Pediatrics. (2017) 140(2):e20163870. 10.1542/peds.2016-3870 28710246

[24] Kirton A Westmacott R deVeber G. Pediatric stroke: rehabilitation of focal injury in the developing brain. NeuroRehabilitation. (2007) 22(5):371–82. 18162700

[25] Lynch JK Hirtz DG DeVeber G Nelson KB. Report of the national institute of neurological disorders and stroke workshop on perinatal and childhood stroke. Pediatrics. (2002) 109(1):116–23. 10.1542/peds.109.1.116 11773550

[26] Goeggel Simonetti B Mono ML Huynh-Do U Michel P Odier C Sztajzel R Risk factors, aetiology and outcome of ischaemic stroke in young adults: the Swiss young stroke study (SYSS). J Neurol. (2015) 262(9):2025–32. 10.1007/s00415-015-7805-5 26067218

[27] Steinlin M Roellin K Schroth G. Long-term follow-up after stroke in childhood. Eur J Pediatr. (2004) 163(4-5):245–50. 10.1007/s00431-003-1357-x 14986120

[28] deVeber GA Kirton A Booth FA Yager JY Wirrell EC Wood E Epidemiology and outcomes of arterial ischemic stroke in children: the Canadian pediatric ischemic stroke registry. Pediatr Neurol. (2017) 69:58–70. 10.1016/j.pediatrneurol.2017.01.016 28254555

[29] Ganesan V Hogan A Shack N Gordon A Isaacs E Kirkham FJ. Outcome after ischaemic stroke in childhood. Dev Med Child Neurol. (2000) 42(7):455–61. 10.1111/j.1469-8749.2000.tb00348.x 10972417

[30] De Schryver EL Kappelle LJ Jennekens-Schinkel A Boudewyn Peters AC. Prognosis of ischemic stroke in childhood: a long-term follow-up study. Dev Med Child Neurol. (2000) 42(5):313–8. 10.1017/s0012162200000554 10855651

[31] Pavlovic J Kaufmann F Boltshauser E Capone Mori A Gubser Mercati D Haenggeli CA Neuropsychological problems after paediatric stroke: two year follow-up of Swiss children. Neuropediatrics. (2006) 37(1):13–9. 10.1055/s-2006-923932 16541363

[32] Max JE Mathews K Lansing AE Robertson BA Fox PT Lancaster JL Psychiatric disorders after childhood stroke. J Am Acad Child Adolesc Psychiatry. (2002) 41(5):555–62. 10.1097/00004583-200205000-00013 12014788

[33] Abgottspon S Thaqi Q Steiner L Slavova N Grunt S Steinlin M Effect of age at pediatric stroke on long-term cognitive outcome. Neurology. (2022) 98(7):e721–e9. 10.1212/WNL.0000000000013207 34916279 PMC8865894

[34] Abgottspon S Steiner L Slavova N Steinlin M Grunt S Everts R. Relationship between motor abilities and executive functions in patients after pediatric stroke. Appl Neuropsychol Child. (2022) 11(4):618–28. 10.1080/21622965.2021.1919111 34043930

[35] McFie J. Intellectual impairment in children with localized post-infantile cerebral lesions. J Neurol Neurosurg Psychiatry. (1961) 24(4):361–5. 10.1136/jnnp.24.4.361 21610903 PMC495398

[36] Banich MT Levine SC Kim H Huttenlocher P. The effects of developmental factors on IQ in hemiplegic children. Neuropsychologia. (1990) 28(1):35–47. 10.1016/0028-3932(90)90084-2 2314563

[37] Jiang B Hills NK Forsyth R Jordan LC Slim M Pavlakis SG Imaging predictors of neurologic outcome after pediatric arterial ischemic stroke. Stroke. (2021) 52(1):152–61. 10.1161/STROKEAHA.120.030965 33280552 PMC7769865

[38] Goeggel Simonetti B Rafay MF Chung M Lo WD Beslow LA Billinghurst LL Comparative study of posterior and anterior circulation stroke in childhood: results from the international pediatric stroke study. Neurology. (2020) 94(4):e337–e44. 10.1212/WNL.0000000000008837 31857436 PMC7978473

[39] Goldenberg NA Jenkins S Jack J Armstrong-Wells J Fenton LZ Stence NV Arteriopathy, D-dimer, and risk of poor neurologic outcome in childhood-onset arterial ischemic stroke. J Pediatr. (2013) 162(5):1041–-6.e1. 10.1016/j.jpeds.2012.11.035 23260102 PMC4115645

[40] Elbers J deVeber G Pontigon AM Moharir M. Long-term outcomes of pediatric ischemic stroke in adulthood. J Child Neurol. (2014) 29(6):782–8. 10.1177/0883073813484358 23589374

[41] Bitsko RH Claussen AH Lichstein J Black LI Jones SE Danielson ML Mental health surveillance among children - United States, 2013–2019. MMWR Suppl. (2022) 71(2):1–42. 10.15585/mmwr.su7102a1 35202359 PMC8890771

[42] Anderson PJ De Luca CR Hutchinson E Roberts G Doyle LW. Victorian infant collaborative G. Underestimation of developmental delay by the new bayley-III scale. Arch Pediatr Adolesc Med. (2010) 164(4):352–6. 10.1001/archpediatrics.2010.20 20368488

[43] Greenham M Gordon A Anderson V Mackay MT. Outcome in childhood stroke. Stroke. (2016) 47(4):1159–64. 10.1161/STROKEAHA.115.011622 26956257

[44] Westmacott R MacGregor D Askalan R deVeber G. Late emergence of cognitive deficits after unilateral neonatal stroke. Stroke. (2009) 40(6):2012–9. 10.1161/STROKEAHA.108.533976 19423855

[45] Farag HF Abdel-Fattah MM Youssri AM. Epidemiological, clinical and prognostic profile of acute bacterial meningitis among children in alexandria, Egypt. Indian J Med Microbiol. (2005) 23(2):95–101. 10.1016/S0255-0857(21)02647-5 15928437

[46] Saha SK Khan NZ Ahmed AS Amin MR Hanif M Mahbub M Neurodevelopmental sequelae in pneumococcal meningitis cases in Bangladesh: a comprehensive follow-up study. Clin Infect Dis. (2009) 48(Suppl 2):S90–6. 10.1086/596545 19191624

[47] Bargui F D'Agostino I Mariani-Kurkdjian P Alberti C Doit C Bellier N Factors influencing neurological outcome of children with bacterial meningitis at the emergency department. Eur J Pediatr. (2012) 171(9):1365–71. 10.1007/s00431-012-1733-5 22527566

[48] Namani S Milenkovic Z Koci B. A prospective study of risk factors for neurological complications in childhood bacterial meningitis. J Pediatr (Rio J). (2013) 89(3):256–62. 10.1016/j.jped.2012.10.001 23664199

[49] Peltola H Roine I Kallio M Pelkonen T. Outcome of childhood bacterial meningitis on three continents. Sci Rep. (2021) 11(1). 10.1038/s41598-021-01085-w PMC8566566 34732790

[50] Kaaresen PI Flaegstad T. Prognostic factors in childhood bacterial meningitis. Acta Paediatr. (1995) 84(8):873–8. 10.1111/j.1651-2227.1995.tb13783.x 7488809

[51] Roine I Peltola H Fernandez J Zavala I Gonzalez Mata A Gonzalez Ayala S Influence of admission findings on death and neurological outcome from childhood bacterial meningitis. Clin Infect Dis. (2008) 46(8):1248–52. 10.1086/533448 18444863

[52] Casella EB Cypel S Osmo AA Okay Y Lefevre BH Lichtig I Sequelae from meningococcal meningitis in children: a critical analysis of dexamethasone therapy. Arq Neuropsiquiatr. (2004) 62(2B):421–8. 10.1590/S0004-282X2004000300009 15273838

[53] Rao S Elkon B Flett KB Moss AF Bernard TJ Stroud B Long-term outcomes and risk factors associated with acute encephalitis in children. J Pediatric Infect Dis Soc. (2017) 6(1):20–7. 10.1093/jpids/piv075 26553786

[54] Koomen I Raat H Jennekens-Schinkel A Grobbee DE Roord JJ van Furth M. Academic and behavioral limitations and health-related quality of life in school-age survivors of bacterial meningitis. Qual Life Res. (2005) 14(6):1563–72. 10.1007/s11136-004-7706-z 16110936

[55] Roed C Omland LH Skinhoj P Rothman KJ Sorensen HT Obel N. Educational achievement and economic self-sufficiency in adults after childhood bacterial meningitis. JAMA. (2013) 309(16):1714–21. 10.1001/jama.2013.3792 23613076

[56] Huang MC Wang SM Hsu YW Lin HC Chi CY Liu CC. Long-term cognitive and motor deficits after enterovirus 71 brainstem encephalitis in children. Pediatrics. (2006) 118(6):e1785–8. 10.1542/peds.2006-1547 17116698

[57] Rautonen J Koskiniemi M Vaheri A. Prognostic factors in childhood acute encephalitis. Pediatr Infect Dis J. (1991) 10(6):441–6. 10.1097/00006454-199106000-00005 1906597

[58] Chin RF Neville BG Peckham C Bedford H Wade A Scott RC Incidence, cause, and short-term outcome of convulsive status epilepticus in childhood: prospective population-based study. Lancet. (2006) 368(9531):222–9. 10.1016/S0140-6736(06)69043-0 16844492

[59] Riney CJ Harding B Harkness WJ Scott RC Cross JH. Hippocampal sclerosis in children with lesional epilepsy is influenced by age at seizure onset. Epilepsia. (2006) 47(1):159–66. 10.1111/j.1528-1167.2006.00382.x 16417544

[60] Cormack F Cross JH Isaacs E Harkness W Wright I Vargha-Khadem F The development of intellectual abilities in pediatric temporal lobe epilepsy. Epilepsia. (2007) 48(1):201–4. 10.1111/j.1528-1167.2006.00904.x 17241230

[61] Scott RC. Adverse outcomes following convulsive status epilepticus in children: relationship with hippocampal injury. Epilepsia. (2010) 51(Suppl 3):178–81. 10.1111/j.1528-1167.2010.02636.x 20618427

[62] Yoong M Martinos MM Chin RF Clark CA Scott RC. Hippocampal volume loss following childhood convulsive status epilepticus is not limited to prolonged febrile seizures. Epilepsia. (2013) 54(12):2108–15. 10.1111/epi.12426 24304434 PMC4377099

[63] Yoong M Seunarine K Martinos M Chin RF Clark CA Scott RC. Prolonged febrile seizures cause reversible reductions in white matter integrity. Neuroimage Clin. (2013) 3:515–21. 10.1016/j.nicl.2013.10.010 24273734 PMC3830064

[64] Jafarpour S Stredny CM Piantino J Chapman KE. Baseline and outcome assessment in pediatric status epilepticus. Seizure. (2019) 68:52–61. 10.1016/j.seizure.2018.04.019 29747930

[65] Nishiyama I Ohtsuka Y Tsuda T Inoue H Kunitomi T Shiraga H An epidemiological study of children with status epilepticus in Okayama, Japan. Epilepsia. (2007) 48(6):1133–7. 10.1111/j.1528-1167.2007.01106.x 17441990

[66] Sillanpaa M Shinnar S. SUDEP And other causes of mortality in childhood-onset epilepsy. Epilepsy Behav. (2013) 28(2):249–55. 10.1016/j.yebeh.2013.04.016 23746924

[67] Lambrechtsen FA Buchhalter JR. Aborted and refractory status epilepticus in children: a comparative analysis. Epilepsia. (2008) 49(4):615–25. 10.1111/j.1528-1167.2007.01465.x 18093148

[68] Kerr C Nixon A Angalakuditi M. The impact of epilepsy on children and adult patients’ lives: development of a conceptual model from qualitative literature. Seizure. (2011) 20(10):764–74. 10.1016/j.seizure.2011.07.007 21831672

[69] Asadi-Pooya AA Poordast A. Etiologies and outcomes of status epilepticus in children. Epilepsy Behav. (2005) 7(3):502–5. 10.1016/j.yebeh.2005.07.005 16146707

[70] Visudtibhan A Limhirun J Chiemchanya S Visudhiphan P. Convulsive status epilepticus in Thai children at ramathibodi hospital. J Med Assoc Thai. (2006) 89(6):803–8. 16850680

[71] Halawa EF Draz I Ahmed D Shaheen HA. Predictors of outcome of convulsive Status epilepticus among an Egyptian pediatric tertiary hospital. J Child Neurol. (2015) 30(13):1736–42. 10.1177/0883073815579706 25895912

[72] Tiwari R Chakrabarty B Gulati S Jauhari P Lodha R Sankar J Development of a novel outcome prediction score (PEDSS) for pediatric convulsive status epilepticus: a longitudinal observational study. Epilepsia. (2020) 61(12):2763–73. 10.1111/epi.16747 33188527

[73] Abend NS Wagenman KL Blake TP Schultheis MT Radcliffe J Berg RA Electrographic status epilepticus and neurobehavioral outcomes in critically ill children. Epilepsy Behav. (2015) 49:238–44. 10.1016/j.yebeh.2015.03.013 25908325 PMC4536172

[74] Topjian AA Gutierrez-Colina AM Sanchez SM Berg RA Friess SH Dlugos DJ Electrographic status epilepticus is associated with mortality and worse short-term outcome in critically ill children. Crit Care Med. (2013) 41(1):215–23. 10.1097/CCM.0b013e3182668035 23164815 PMC3531581

[75] Jayaram N McNally B Tang F Chan PS. Survival after out-of-hospital cardiac arrest in children. J Am Heart Assoc. (2015) 4(10):e002122. 10.1161/JAHA.115.002122 26450118 PMC4845116

[76] Yan S Gan Y Jiang N Wang R Chen Y Luo Z The global survival rate among adult out-of-hospital cardiac arrest patients who received cardiopulmonary resuscitation: a systematic review and meta-analysis. Crit Care. (2020) 24(1):61. 10.1186/s13054-020-2773-2 32087741 PMC7036236

[77] Holmberg MJ Wiberg S Ross CE Kleinman M Hoeyer-Nielsen AK Donnino MW Trends in survival after pediatric in-hospital cardiac arrest in the United States. Circulation. (2019) 140(17):1398–408. 10.1161/CIRCULATIONAHA.119.041667 31542952 PMC6803102

[78] Atkins DL Everson-Stewart S Sears GK Daya M Osmond MH Warden CR Epidemiology and outcomes from out-of-hospital cardiac arrest in children: the resuscitation outcomes consortium epistry-cardiac arrest. Circulation. (2009) 119(11):1484–91. 10.1161/CIRCULATIONAHA.108.802678 19273724 PMC2679169

[79] Knudson JD Neish SR Cabrera AG Lowry AW Shamszad P Morales DL Prevalence and outcomes of pediatric in-hospital cardiopulmonary resuscitation in the United States: an analysis of the Kids’ inpatient database*. Crit Care Med. (2012) 40(11):2940–4. 10.1097/CCM.0b013e31825feb3f 22932398

[80] Virani SS Alonso A Benjamin EJ Bittencourt MS Callaway CW Carson AP Heart disease and stroke statistics-2020 update: a report from the American Heart Association. Circulation. (2020) 141(9):e139–596. 10.1161/CIR.0000000000000757 31992061

[81] Lee EP Chan OW Lin JJ Hsia SH Wu HP. Risk factors and neurologic outcomes associated with resuscitation in the pediatric intensive care unit. Front Pediatr. (2022) 10:834746. 10.3389/fped.2022.834746 35444968 PMC9013941

[82] Michiels E Quan L Dumas F Rea T. Long-term neurologic outcomes following paediatric out-of-hospital cardiac arrest. Resuscitation. (2016) 102:122–6. 10.1016/j.resuscitation.2016.01.010 26826563

[83] Kriel RL Krach LE Luxenberg MG Jones-Saete C Sanchez J. Outcome of severe anoxic/ischemic brain injury in children. Pediatr Neurol. (1994) 10(3):207–12. 10.1016/0887-8994(94)90024-8 8060422

[84] Maryniak A Bielawska A Walczak F Szumowski L Bieganowska K Rekawek J Long-term cognitive outcome in teenage survivors of arrhythmic cardiac arrest. Resuscitation. (2008) 77(1):46–50. 10.1016/j.resuscitation.2007.10.024 18207629

[85] van Zellem L Utens EM Legerstee JS Cransberg K Hulst JM Tibboel D Cardiac arrest in children: long-term health Status and health-related quality of life. Pediatr Crit Care Med. (2015) 16(8):693–702. 10.1097/PCC.0000000000000452 26020858

[86] van Zellem L Buysse C Madderom M Legerstee JS Aarsen F Tibboel D Long-term neuropsychological outcomes in children and adolescents after cardiac arrest. Intensive Care Med. (2015) 41(6):1057–66. 10.1007/s00134-015-3789-y 25894622 PMC4477720

[87] Li G Tang N DiScala C Meisel Z Levick N Kelen GD. Cardiopulmonary resuscitation in pediatric trauma patients: survival and functional outcome. J Trauma. (1999) 47(1):1–7. 10.1097/00005373-199907000-00001 10421178

[88] Suominen PK Sutinen N Valle S Olkkola KT Lonnqvist T. Neurocognitive long term follow-up study on drowned children. Resuscitation. (2014) 85(8):1059–64. 10.1016/j.resuscitation.2014.03.307 24709615

[89] Horisberger T Fischer E Fanconi S. One-year survival and neurological outcome after pediatric cardiopulmonary resuscitation. Intensive Care Med. (2002) 28(3):365–8. 10.1007/s00134-001-1188-z 11904669

[90] Slomine BS Silverstein FS Christensen JR Holubkov R Telford R Dean JM Neurobehavioural outcomes in children after in-hospital cardiac arrest. Resuscitation. (2018) 124:80–9. 10.1016/j.resuscitation.2018.01.002 29305927 PMC5837951

[91] Albrecht M de Jonge RCJ Nadkarni VM de Hoog M Hunfeld M Kammeraad JAE Association between shockable rhythms and long-term outcome after pediatric out-of-hospital cardiac arrest in rotterdam, The Netherlands: an 18-year observational study. Resuscitation. (2021) 166:110–20. 10.1016/j.resuscitation.2021.05.015 34082030

[92] Hunfeld M Dulfer K Rietman A Pangalila R van Gils-Frijters A Catsman-Berrevoets C Longitudinal two years evaluation of neuropsychological outcome in children after out of hospital cardiac arrest. Resuscitation. (2021) 167:29–37. 10.1016/j.resuscitation.2021.07.043 34389455

[93] Hickson MR Winters M Thomas NH Gardner MM Kirschen MP Nadkarni V Long-term function, quality of life and healthcare utilization among survivors of pediatric out-of-hospital cardiac arrest. Resuscitation. (2023) 187:109768. 10.1016/j.resuscitation.2023.109768 36933881 PMC10267669

[94] Del Castillo J Lopez-Herce J Matamoros M Canadas S Rodriguez-Calvo A Cecchetti C Long-term evolution after in-hospital cardiac arrest in children: prospective multicenter multinational study. Resuscitation. (2015) 96:126–34. 10.1016/j.resuscitation.2015.07.037 26296583

[95] Fink EL Berger RP Clark RS Watson RS Angus DC Richichi R Serum biomarkers of brain injury to classify outcome after pediatric cardiac arrest*. Crit Care Med. (2014) 42(3):664–74. 10.1097/01.ccm.0000435668.53188.80 24164954 PMC4478619

[96] Fink EL Kochanek PM Panigrahy A Beers SR Berger RP Bayir H Association of blood-based brain injury biomarker concentrations with outcomes after pediatric cardiac arrest. JAMA Netw Open. (2022) 5(9):e2230518. 10.1001/jamanetworkopen.2022.30518 36074465 PMC9459665

[97] Topjian AA Lin R Morris MC Ichord R Drott H Bayer CR Neuron-specific enolase and S-100B are associated with neurologic outcome after pediatric cardiac arrest. Pediatr Crit Care Med. (2009) 10(4):479–90. 10.1097/PCC.0b013e318198bdb5 19307814

[98] Valles-Colomer M Blanco-Miguez A Manghi P Asnicar F Dubois L Golzato D The person-to-person transmission landscape of the gut and oral microbiomes. Nature. (2023) 614(7946):125–35. 10.1038/s41586-022-05620-1 36653448 PMC9892008

[99] Chu DM Ma J Prince AL Antony KM Seferovic MD Aagaard KM. Maturation of the infant microbiome community structure and function across multiple body sites and in relation to mode of delivery. Nat Med. (2017) 23(3):314–26. 10.1038/nm.4272 28112736 PMC5345907

[100] Hill CJ Lynch DB Murphy K Ulaszewska M Jeffery IB O’Shea CA Evolution of gut microbiota composition from birth to 24 weeks in the INFANTMET cohort. Microbiome. (2017) 5(1):4. 10.1186/s40168-016-0213-y 28095889 PMC5240274

[101] Stewart CJ Ajami NJ O’Brien JL Hutchinson DS Smith DP Wong MC Temporal development of the gut microbiome in early childhood from the TEDDY study. Nature. (2018) 562(7728):583–8. 10.1038/s41586-018-0617-x 30356187 PMC6415775

[102] Hopkins MJ Sharp R Macfarlane GT. Variation in human intestinal microbiota with age. Dig Liver Dis. (2002) 34(Suppl 2):S12–8. 10.1016/S1590-8658(02)80157-8 12408433

[103] Agans R Rigsbee L Kenche H Michail S Khamis HJ Paliy O. Distal gut microbiota of adolescent children is different from that of adults. FEMS Microbiol Ecol. (2011) 77(2):404–12. 10.1111/j.1574-6941.2011.01120.x 21539582 PMC4502954

[104] Rogers MB Simon D Firek B Silfies L Fabio A Bell MJ Temporal and spatial changes in the microbiome following pediatric severe traumatic brain injury. Pediatr Crit Care Med. (2022) 23(6):425–34. 10.1097/PCC.0000000000002929 35283451 PMC9203870

[105] Rogers MB Firek B Shi M Yeh A Brower-Sinning R Aveson V Disruption of the microbiota across multiple body sites in critically ill children. Microbiome. (2016) 4(1):66. 10.1186/s40168-016-0211-0 28034303 PMC5200963

[106] Kitsios GD Morowitz MJ Dickson RP Huffnagle GB McVerry BJ Morris A. Dysbiosis in the intensive care unit: microbiome science coming to the bedside. J Crit Care. (2017) 38:84–91. 10.1016/j.jcrc.2016.09.029 27866110 PMC5328797

[107] McDonnell L Gilkes A Ashworth M Rowland V Harries TH Armstrong D Association between antibiotics and gut microbiome dysbiosis in children: systematic review and meta-analysis. Gut Microbes. (2021) 13(1):1–18. 10.1080/19490976.2020.1870402 33651651 PMC7928022

[108] Becattini S Taur Y Pamer EG. Antibiotic-Induced changes in the intestinal Microbiota and disease. Trends Mol Med. (2016) 22(6):458–78. 10.1016/j.molmed.2016.04.003 27178527 PMC4885777

[109] David LA Materna AC Friedman J Campos-Baptista MI Blackburn MC Perrotta A Host lifestyle affects human microbiota on daily timescales. Genome Biol. (2014) 15(7):R89. 10.1186/gb-2014-15-7-r89 25146375 PMC4405912

[110] Thaiss CA Itav S Rothschild D Meijer MT Levy M Moresi C Persistent microbiome alterations modulate the rate of post-dieting weight regain. Nature. (2016) 540(7634):544–51. 10.1038/nature20796 27906159

[111] Fonseca DM Hand TW Han SJ Gerner MY Glatman Zaretsky A Byrd AL Microbiota-dependent sequelae of acute infection compromise tissue-specific immunity. Cell. (2015) 163(2):354–66. 10.1016/j.cell.2015.08.030 26451485 PMC4826740

[112] Ronan V Yeasin R Claud EC. Childhood development and the microbiome—the intestinal Microbiota in maintenance of health and development of disease during childhood development. Gastroenterology. (2021) 160(2):495–506. 10.1053/j.gastro.2020.08.065 33307032 PMC8714606

[113] Simon DW Rogers MB Gao Y Vincent G Firek BA Janesko-Feldman K Depletion of gut microbiota is associated with improved neurologic outcome following traumatic brain injury. Brain Res. (2020) 1747:147056. 10.1016/j.brainres.2020.147056 32798452 PMC7521107

[114] Yeh A Rogers MB Firek B Neal MD Zuckerbraun BS Morowitz MJ. Dysbiosis across multiple body sites in critically ill adult surgical patients. Shock. (2016) 46(6):649–54. 10.1097/SHK.0000000000000691 27454385

[115] Treangen TJ Wagner J Burns MP Villapol S. Traumatic brain injury in mice induces acute bacterial dysbiosis within the fecal microbiome. Front Immunol. (2018) 9:2757. 10.3389/fimmu.2018.02757 30546361 PMC6278748

[116] Opeyemi OM Rogers MB Firek BA Janesko-Feldman K Vagni V Mullett SJ Sustained dysbiosis and decreased fecal short-chain fatty acids after traumatic brain injury and impact on neurologic outcome. J Neurotrauma. (2021) 38(18):2610–21. 10.1089/neu.2020.7506 33957773 PMC8403202

[117] Koh A De Vadder F Kovatcheva-Datchary P Backhed F. From dietary fiber to host physiology: short-chain fatty acids as key bacterial metabolites. Cell. (2016) 165(6):1332–45. 10.1016/j.cell.2016.05.041 27259147

[118] Dalile B Vervliet B Bergonzelli G Verbeke K Van Oudenhove L. Colon-delivered short-chain fatty acids attenuate the cortisol response to psychosocial stress in healthy men: a randomized, placebo-controlled trial. Neuropsychopharmacology. (2020) 45(13):2257–66. 10.1038/s41386-020-0732-x 32521538 PMC7784980

[119] van de Wouw M Boehme M Lyte JM Wiley N Strain C O’Sullivan O Short-chain fatty acids: microbial metabolites that alleviate stress-induced brain-gut axis alterations. J Physiol. (2018) 596(20):4923–44. 10.1113/JP276431 30066368 PMC6187046

[120] Strandwitz P. Neurotransmitter modulation by the gut microbiota. Brain Res. (2018) 1693(Pt B):128–33. 10.1016/j.brainres.2018.03.015 29903615 PMC6005194

[121] Galland L. The gut microbiome and the brain. J Med Food. (2014) 17(12):1261–72. 10.1089/jmf.2014.7000 25402818 PMC4259177

[122] Del Colle A Israelyan N Gross Margolis K. Novel aspects of enteric serotonergic signaling in health and brain-gut disease. Am J Physiol Gastrointest Liver Physiol. (2020) 318(1):G130–G43. 10.1152/ajpgi.00173.2019 31682158 PMC6985840

[123] Smith D Jheeta S Fuentes HV Palacios-Perez M. Feeding our Microbiota: stimulation of the immune/semiochemical system and the potential amelioration of non-communicable diseases. Life (Basel). (2022) 12(8):1197. 10.3390/life12081197 36013376 PMC9410320

[124] Aarts E Ederveen THA Naaijen J Zwiers MP Boekhorst J Timmerman HM Gut microbiome in ADHD and its relation to neural reward anticipation. PLoS One. (2017) 12(9):e0183509. 10.1371/journal.pone.0183509 28863139 PMC5581161

[125] Carlson AL Xia K Azcarate-Peril MA Goldman BD Ahn M Styner MA Infant gut microbiome associated with cognitive development. Biol Psychiatry. (2018) 83(2):148–59. 10.1016/j.biopsych.2017.06.021 28793975 PMC5724966

[126] Magula L Moxley K Lachman A. Iron deficiency in South African children and adolescents with attention deficit hyperactivity disorder. J Child Adolesc Ment Health. (2019) 31(2):85–92. 10.2989/17280583.2019.1637345 31339453

[127] Stewart A Davis GL Gresch PJ Katamish RM Peart R Rabil MJ Serotonin transporter inhibition and 5-HT(2C) receptor activation drive loss of cocaine-induced locomotor activation in DAT Val559 mice. Neuropsychopharmacology. (2019) 44(5):994–1006. 10.1038/s41386-018-0301-8 30578419 PMC6462012

[128] Suzuki C Ikeda Y Tateno A Okubo Y Fukayama H Suzuki H. Acute atomoxetine selectively modulates encoding of reward value in ventral medial prefrontal cortex. J Nippon Med Sch. (2019) 86(2):98–107. 10.1272/jnms.JNMS.2019_86-205 31130571

[129] Ihekweazu FD Versalovic J. Development of the pediatric gut microbiome: impact on health and disease. Am J Med Sci. (2018) 356(5):413–23. 10.1016/j.amjms.2018.08.005 30384950 PMC6268214

[130] Sharon G Cruz NJ Kang DW Gandal MJ Wang B Kim YM Human gut Microbiota from autism spectrum disorder promote behavioral symptoms in mice. Cell. (2019) 177(6):1600–18.e17. 10.1016/j.cell.2019.05.004 31150625 PMC6993574

[131] Zhang Y Zhang J Pan Z He X. Effects of washed fecal bacteria transplantation in sleep quality, stool features and autism symptomatology: a Chinese preliminary observational study. Neuropsychiatr Dis Treat. (2022) 18:1165–73. 10.2147/NDT.S355233 35719863 PMC9199912

[132] Li N Chen H Cheng Y Xu F Ruan G Ying S Corrigendum: fecal Microbiota transplantation relieves gastrointestinal and autism symptoms by improving the gut Microbiota in an open-label study. Front Cell Infect Microbiol. (2021) 11:801376. 10.3389/fcimb.2021.801376 34888262 PMC8650714

[133] Kang DW Adams JB Gregory AC Borody T Chittick L Fasano A Microbiota transfer therapy alters gut ecosystem and improves gastrointestinal and autism symptoms: an open-label study. Microbiome. (2017) 5(1):10. 10.1186/s40168-016-0225-7 28122648 PMC5264285

[134] Bravo JA Forsythe P Chew MV Escaravage E Savignac HM Dinan TG Ingestion of Lactobacillus strain regulates emotional behavior and central GABA receptor expression in a mouse via the vagus nerve. Proc Natl Acad Sci U S A. (2011) 108(38):16050–5. 10.1073/pnas.1102999108 21876150 PMC3179073

[135] Diaz Heijtz R Wang S Anuar F Qian Y Bjorkholm B Samuelsson A Normal gut microbiota modulates brain development and behavior. Proc Natl Acad Sci U S A. (2011) 108(7):3047–52. 10.1073/pnas.1010529108 21282636 PMC3041077

[136] Valles-Colomer M Falony G Darzi Y Tigchelaar EF Wang J Tito RY The neuroactive potential of the human gut microbiota in quality of life and depression. Nat Microbiol. (2019) 4(4):623–32. 10.1038/s41564-018-0337-x 30718848

[137] Semple BD Blomgren K Gimlin K Ferriero DM Noble-Haeusslein LJ. Brain development in rodents and humans: identifying benchmarks of maturation and vulnerability to injury across species. Prog Neurobiol. (2013) 106-107:1–16. 10.1016/j.pneurobio.2013.04.001 23583307 PMC3737272

[138] Dennis M. Margaret Kennard (1899–1975): not a ‘principle’ of brain plasticity but a founding mother of developmental neuropsychology. Cortex. (2010) 46(8):1043–59. 10.1016/j.cortex.2009.10.008 20079891 PMC2907425

[139] Morris RG. D.O. Hebb: the Organization of Behavior, Wiley: New York; 1949. Brain Res Bull. (1999) 50(5-6):437–00. 10.1016/s0361-9230(99)00182-3 10643472

[140] Krageloh-Mann I Lidzba K Pavlova MA Wilke M Staudt M. Plasticity during early brain development is determined by ontogenetic potential. Neuropediatrics. (2017) 48(2):66–71. 10.1055/s-0037-1599234 28282668

[141] Lidzba K Wilke M Staudt M Krageloh-Mann I. Early plasticity versus early vulnerability: the problem of heterogeneous lesion types. Brain. (2009) 132(Pt 10):e128. author reply e9. 10.1093/brain/awp197 19661250

[142] Lopez-Espejo M Hernandez-Chavez M. Could infarct location predict the long-term functional outcome in childhood arterial ischemic stroke? Arq Neuropsiquiatr. (2017) 75(10):692–6. 10.1590/0004-282x20170124 29166459

[143] Lopez-Espejo M Hernandez-Chavez M. Prevalence and predictors of long-term functional impairment, epilepsy, mortality, and stroke recurrence after childhood stroke: a prospective study of a Chilean cohort. J Stroke Cerebrovasc Dis. (2017) 26(7):1646–52. 10.1016/j.jstrokecerebrovasdis.2017.03.043 28476510

[144] Anderson V Catroppa C Morse S Haritou F Rosenfeld J. Functional plasticity or vulnerability after early brain injury? Pediatrics. (2005) 116(6):1374–82. 10.1542/peds.2004-1728 16322161

[145] Anderson V Jacobs R Spencer-Smith M Coleman L Anderson P Williams J Does early age at brain insult predict worse outcome? Neuropsychological implications. J Pediatr Psychol. (2010) 35(7):716–27. 10.1093/jpepsy/jsp100 19995865

[146] Anderson V Spencer-Smith M Wood A. Do children really recover better? Neurobehavioural plasticity after early brain insult. Brain. (2011) 134(Pt 8):2197–221. 10.1093/brain/awr103 21784775

[147] Karver CL Wade SL Cassedy A Taylor HG Stancin T Yeates KO Age at injury and long-term behavior problems after traumatic brain injury in young children. Rehabil Psychol. (2012) 57(3):256–65. 10.1037/a0029522 22946613 PMC3750969

[148] Cowan CSM Dinan TG Cryan JF. Annual research review: critical windows—the microbiota–gut–brain axis in neurocognitive development. J Child Psychol Psychiatry. (2020) 61(3):353–71. 10.1111/jcpp.13156 31773737

[149] Lynch CMK Cowan CSM Bastiaanssen TFS Moloney GM Theune N van de Wouw M Critical windows of early-life microbiota disruption on behaviour, neuroimmune function, and neurodevelopment. Brain Behav Immun. (2023) 108:309–27. 10.1016/j.bbi.2022.12.008 36535610

[150] Callaghan B. Commentary: microbial panaceas: does development have the answer? - reflections on cowan, dinan, & cryan (2020). J Child Psychol Psychiatry. (2020) 61(3):372–5. 10.1111/jcpp.13192 31944315 PMC8009034

[151] Slykerman RF Thompson J Waldie KE Murphy R Wall C Mitchell EA. Antibiotics in the first year of life and subsequent neurocognitive outcomes. Acta Paediatr. (2017) 106(1):87–94. 10.1111/apa.13613 27701771

[152] Nelson C. The neurobiological bases of early intervention. In: Shonkoff JP MS, editor. Handbook of Early Childhood Intervention. Cambridge University Press (2000). p. 204–28.

